# MAO-A Inhibitory Potential of Terpene Constituents from Ginger Rhizomes—A Bioactivity Guided Fractionation

**DOI:** 10.3390/molecules23061301

**Published:** 2018-05-29

**Authors:** Wirginia Kukula-Koch, Wojciech Koch, Lidia Czernicka, Kazimierz Głowniak, Yoshinori Asakawa, Akemi Umeyama, Zbigniew Marzec, Takashi Kuzuhara

**Affiliations:** 1 Chair and Department of Pharmacognosy with Medical Plant Unit, Medical University of Lublin, 1 Chodźki Str., 20-093 Lublin, Poland; kglowniak@pharmacognosy.org; 2 Chair and Department of Food and Nutrition, Medical University of Lublin, 4a Chodźki Str., 20-093 Lublin, Poland; kochw@interia.pl (W.K.); lidia.czernicka@umlub.pl (L.C.); zbigniew.marzec@umlub.pl (Z.M.); 3 Department of Cosmetology, University of Information Technology and Management in Rzeszów, Kielnarowa 386a, 36-020 Tyczyn, Poland; 4 Department of Pharmaceutical Chemistry, Faculty of Pharmaceutical Sciences, Tokushima Bunri University, Yamashiro-cho, Tokushima 770-8514, Japan; asakawa@ph.bunri-u.ac.jp; 5 Department of Pharmacognosy, Faculty of Pharmaceutical Sciences, Tokushima Bunri University, Yamashiro-cho, Tokushima 770-8514, Japan; umeyama@ph.bunri-u.ac.jp; 6 Laboratory of Biochemistry, Faculty of Pharmaceutical Sciences, Tokushima Bunri University, Yamashiro-cho, Tokushima 770-8514, Japan; kuzuhara@ph.bunri-u.ac.jp

**Keywords:** *Zingiber officinale*, Zingiberaceae, MAO-A inhibition, depression, antidepressants, terpenes

## Abstract

Background: In the search for novel antidepressive drug candidates, bioguided fractionation of nonpolar constituents present in the oleoresin from ginger rhizomes (*Zingiber officinale* Roscoe, Zingiberaceae) was performed. This particular direction of the research was chosen due to the existing reports on the antidepressive properties of ginger total extract. The search for individual metabolites acting as MAO-A inhibitors, which correspond to the apparent effect of the total extract, is the subject of this work. Methods: Hexane extracts from ginger rhizomes were fractionated by using column chromatography (including silica gel impregnated with silver nitrate) and semi-preparative high-performance chromatography. For the activity assessment, an in vitro monoamine oxidase A (MAO-A) inhibition luminescence assay was performed on 10 purified terpenes: 1,8-cineole, α-citronellal, geraniol, β-sesquiphellandrene, γ-terpinen, geranyl acetate, isobornyl acetate, terpinen-4-ol, (*E*,*E*)-α-farnesene, and α-zingiberene. Results: Geraniol and (−)-terpinen-4-ol were found to be the strongest enzyme inhibitors with inhibition of 44.1% and 42.5%, respectively, at a concentration of 125 µg/mL. No differences in the inhibition potential were observed for the different groups of terpenes: sesquiterpenes, monoterpenes, or sesquiterpene alcohols; however, the two most active compounds contained a hydroxyl moiety. Conclusions: Terpene constituents from ginger’s extract were found to exhibit moderate inhibitory properties against the MAO-A enzyme in in vitro tests.

## 1. Introduction

Ginger (*Zingiber officinale* Roscoe) is one of the most important and most commonly used spices around the world. This plant has a long history of application in medicine, which includes use as treatment against nausea, vomiting, dyspepsia, and loss of appetite, constipation, or indigestion. It is also used in the treatment of inflammation during various diseases, including rheumatism, central nervous system (CNS) disorders, and pain [1]. Ginger has long been used in Hindu, Chinese and Japanese medicine as anti-inflammatory, immunostimulating, antibacterial, anti-atherosclerotic, and anti-migraine drug [1,2]. Its ability to penetrate the blood–brain barrier and elicit pharmacological effects in the CNS, including psychiatric disorders e.g., neurosis, stroke, brain tumors and Alzheimer disease, was confirmed in various pharmacological studies [3]. In addition, this plant, as an edible one, is characterized by low toxicity, which allows higher dosages and so more visible pharmacological effects of the supplementation. Currently, after elucidating some of the steps in the mechanism of action of ginger on the CNS, there is a possibility of using its therapeutic effect in the treatment of depression. Only a few studies were performed to confirm the ability of ginger extracts to inhibit MAO-A enzyme activity and to agonize serotoninergic receptors 5-HT1A/1B/7 [3,4,5]. It is surprising, however, that the vast majority of animal studies include only total extracts obtained from ginger and not the single components. Ginger rhizome is a rich source of bioactive compounds: essential oils (rich in volatile components), phenolic compounds, flavonoids, and alkaloids [6,7]. The phenolic compounds, classified as gingerols and shogaols are the main ingredients that exhibit antioxidant properties in ginger extract and are the main target of scientific interest, regarding their biological properties [8,9].

The main therapeutic strategy in the treatment of depression and mood disorders is still the pharmacological modulation of the monoamine system. Since neurotransmitters (dopamine, norepinephrine and serotonin) are metabolized by monoamine oxidase (MAO), the inhibition of the enzyme may increase their brain concentrations and thus reduce disease symptoms [10,11]. MAO inhibitors (MAOIs) have been used in depressive therapies for over 50 years so far. Because MAO-A mostly metabolize serotonin and norepinephrine, the inhibitors of this enzyme are mainly used in the treatment of depression. Currently selective and reversible MAO-A inhibitors, with moclobemide being the major representative, are administered in the treatment of mental disorders. Its therapeutic efficacy was demonstrated in numerous studies [12,13]. Also, other drugs, such as phenelzine and tranylcypromine are still used in the treatment of atypical depression, bulimia nervosa and panic disorders in the United States [14,15]. Despite their high effectiveness, compared to tricyclic antidepressants (TCAs) and selective serotonin reuptake inhibitors (SSRI), the wide spectrum of side effects and the development of new treatment strategies have meant that these drugs are now not considered first-line drugs [16,17]. MAOIs possess other beneficial effects, including oxidative stress protection [18,19].

Research into better understanding of MAOIs and the development of new inhibitors of this enzyme led to the discovery of several active agents among natural products. Among the available literature there are reports on MAO inhibitory properties of natural compounds from plants such as: *Huperzia brevifolia* and *Huperzia espinosana* [20], *Rhodiola rosea* L. roots [21], *Hypericum androsaemum*, *Caryratia japonica*, *Calluna vulgaris* and *Ginko biloba* [22,23], *Borago officinalis*, *Trigonella foenum-graecum*, *Apium graveolens*, *Calluna vulgaris* [24], *Paeonia moutan* and *Primula auricula* [25]. There are also studies on the activity of pure compounds of natural origin towards MAO enzyme inhibition, for example emodin (from *Polygonum multiflorum*), piperine (*Piper nigrum*) [26], quercetin isolated from *Hypericum thasium* [22] and naringenin (from *Mentha aquatica* L.) [27].

Only few studies have confirmed the ability of ginger extract to inhibit MAO-A and agonize serotoninergic pathways and these data concern whole extracts and not individual active compounds. Recently the results of two different studies [28,29] revealed that polyphenols from ginger are responsible for its MAO-A inhibitory potential, but no data on the lipophilic fraction from *Zingiber officinale* rhizome was reported. Therefore the main objective of the study was to evaluate the potential of the volatile fraction, which is highly represented in this plant, towards inhibition of the MAO-A enzyme. Taking into account that such data have not been published so far, the results of the present study may expand the existing knowledge on the antidepressant potential of ginger and its active compounds.

## 2. Results and Discussion

Depressive disorders constitute a growing problem affecting residents of developed countries all over the world. The development in medicine in the past decades and the rise in patients’ awareness contribute to the prolongation of life. However, this has led to generation of central nervous system (CNS) disorders. It is estimated that the number of people suffering from depression is around 120 million. In light of the above data, depression is commonly referred to as the 21st century epidemic (WHO data).

One of the key requirements of modern research is to increase the effectiveness of existing and developing new therapeutic strategies for the CNS diseases including the serotoninergic and dopaminergic system disorders, which end up causing depression. The currently available medicines are subject to a number of side effects, which include a significant inconvenience—weight gain. This fact limits the use of most commercially available medicinal preparations. Because of this fact, the need for an increased selectivity toward different neurotransmitters is needed in the evaluation of drug candidates to reduce their side effects.

At the moment, the European market for over-the-counter drugs does not have much to offer patients suffering from mild forms of depression. The most commonly administered dietary supplements or OTC drugs contain St. John’s wort or tryptophan as a precursor of neuromodulators which lack in the patients with depression. However, the herb exhibits important side effects in the form of drug interactions or skin photosensitization, which often results in painful sunburns or melanin production disturbances [30].

That is why, the authors found it important to study the potential of some novel drug candidates. Plant secondary metabolites constitute a naturally available library of different organic compounds and are an inspiration for the process of new drugs acquisition; that is why in the described study a known and widely used plant species—*Zingiber officinale*—was evaluated.

### 2.1. Bioguided Fractionation of Ginger Extract

The constituents of ginger’s rhizome may be divided into two major groups: lipids and oleoresins. The latter—rich in secondary metabolites—is composed of terpenes (monoterpenes, sesquiterpenes, and sesquiterpene alcohols) in 20–25% and of phenolics–gingerols (including gingerodiones, gingerdiols, dehydrogingerdiones, and diarylheptanoids), which account for another 25% of the semi-liquid resin [9,31,32].

The application of chromatographic methods—column chromatography and semi-preparative HPLC under optimized conditions enabled the successful fractionation of ginger crude extract into single compounds.

**Figure 1:**
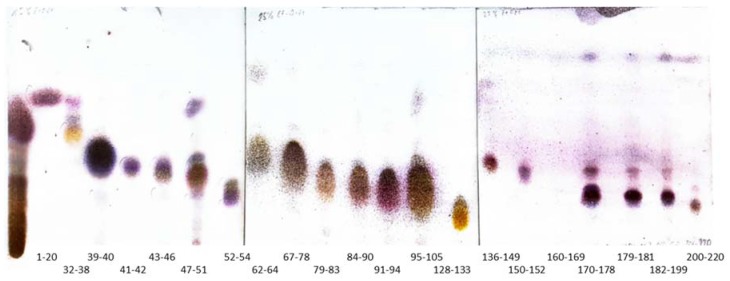
TLC chromatograms (NP Silica Gel TLC plates, Merck, Darmstadt, Germany) obtained for the collective fractions from column 1 in the following solvent systems: 15% of diethyl ether in *n*-hexane (fractions 1–54), 25% of diethyl ether in *n*-hexane (fractions 62–133) and 29% of diethyl ether in *n*-hexane (fractions 136–220) detected by a solution of p-anisaldehyde in methanol.

Column 1—a gravity chromatographic column developed from separation of the total extract of ginger in a gradient of EtOEt in *n*-hexane resulted in a successful fractionation of this complex matrix into fractions which differed in composition between one another. Together 233 fractions were collected, among which 21 collective ones were finally obtained (Figure 1), dried, weighted, and subjected to further analysis.

**Figure 2:**
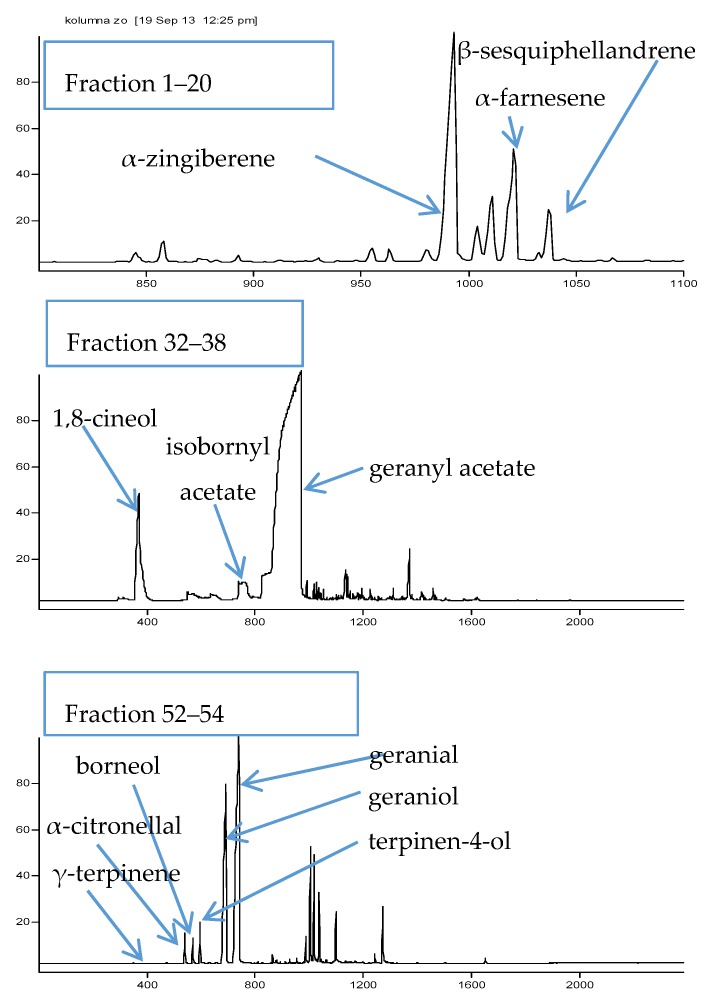
The composition of three most active collective fractions from column 1 determined by GC-MS analysis (y axis—number of scan, x axis—intensity of the peak in relation to the most abundant one determined as 100%).

**Figure 3:**
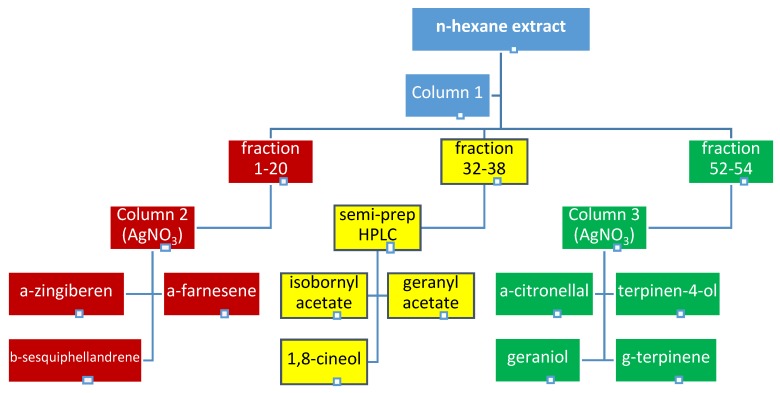
The scheme of bioguided fractionation of the total extract (CC—column chromatography).

In the search for MAO-A inhibitors, an initial screening towards the identification of the most promising MAO-A inhibitors was performed on each obtained collective fraction. Three most active ones (see Figure 2 and Figure 3) were selected for further fractionation and detailed composition analyses. These were: fractions 1–20 with a measured inhibition value of 36.5%, fractions 32–38 of 32.9%, and fractions 52–54 of 37.6%.

A GC-MS based qualitative analysis was performed on the three collective samples prior to further fractionation. Several compounds, which belonged to the group of terpenes were identified based on their retention indices, literature data and previous studies of the authors [32]. The identification data of major constituents are presented in Table 1. All identified compounds were also previously described by other authors as major components of ginger extracts [33]. The obtained spectra showing the qualitative composition of the fractions subjected to further division are presented in the Figure 2.

The successful fractionation of sample containing fractions 1–20 (column 2) and fractions 52–54 (column 3) was possible thanks to the use of silica gel impregnated with silver nitrate, washed with 2% EtOEt in *n*-hexane.

Impregnation of silica gel with silver nitrate provided fast and effective isolation of terpenes, based on their chemical structure. In this instance, the separation of constituents occurred relative to the number of double bonds in a compound, corresponding to the interaction of their π-cleavages with impregnated silica [34]. The more double compounds present in the molecule, the stronger the interaction with adsorbent and thus the elution time is prolonged. As a result completely saturated terpenes were eluted first from the column, later double bond compounds etc. The use of these interaction for separation was helpful in the purification of single terpenes from the matrix due to the presence of structurally very similar compounds in the ginger extract. Since ar-curcumene, α-zingiberene, *ƴ*-bisabolene, or β-sesquiphellandrene differ from one another only in the location and quantity of double bonds, previous attempts of the authors to separate the sesquiterpenes complex on an unmodified adsorbent had not been successful (see Figure S1 Supplementary File).

The development of column 2 provided 113 subfractions and contained purified α-zingiberene (23.88 mg), α-farnesene (11.54 mg) and β-sesquiphellandrene (5.18 mg). A similar adsorbent was used to subfractionate the abovementioned sample on column 3. This time 500 mg of the sample resulted in 55.3 mg of geranial, 33.71 mg of geraniol, 7.43 mg of terpinen-4-ol, 6.92 mg of citronellal and 2.41 mg of γ-terpinene.

Fraction 32–38 was subjected to semi-preparative normal phase HPLC purification in the optimized method and it yielded three major constituents at a purity exceeding 95%: 1,8-cineole (5.61 mg), isobornyl acetate (5.28 mg) and geranyl acetate (13.92 mg).

### 2.2. MAO-A Inhibition Assessment of Purified Terpenes from Ginger Rhizomes

**Figure 4:**
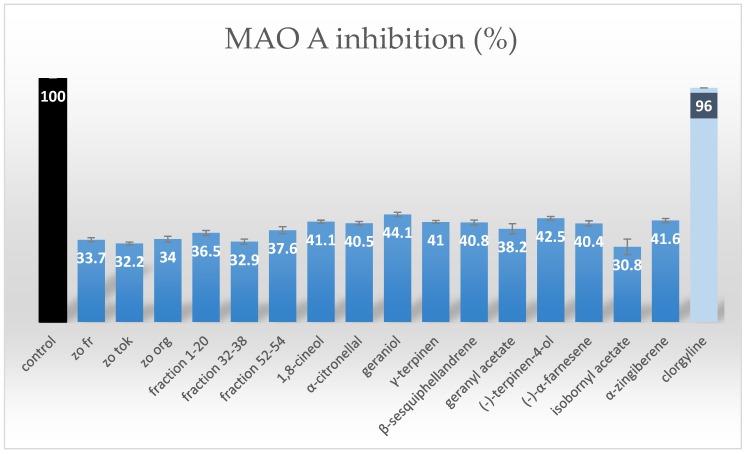
The percentage of MAO-A inhibition with standard deviation (SD) obtained for different ginger extracts (zo fr, zo tok, zo org), selected fractions from column 1 (fraction 0–20, 32–38 and 52–54) and purified terpenes (Control—MAO-A assay performed without any inhibitor shows the maximum of luminescence; clorgyline—the inhibition of a known inhibitor based on the MAO-A assay producer’s information in the same assay).

The percentage of MAO-A inhibition was measured for each isolated compound, according to the described protocol. The obtained results are presented in Figure 4. The graph clearly shows the differences between the activity of the collective fractions and the purified terpenes. The latter were found to exhibit stronger inhibitory activity in comparison with the fractions or with the total extracts. The obtained values of percentage inhibition for single compounds ranged from 30.8% to 44.1%. Among compounds studied, geraniol was found to be the strongest inhibitor and its percentage inhibition was calculated as 44.1%. An acetylation of this terpene led to a statistically significant decrease in the studied activity. What is more, both geranyl acetate and bornyl acetate were the weakest inhibitors, which led to the conclusion that hydroxyl group substitution decreased this biological activity. The percentage inhibition of the enzyme calculated for other studied terpenes remained on a similar level, ranging around 40%, at the test sample concentration of 125 µg/mL. No statistically significant difference between the activity of the various types of terpenes was confirmed, however, two of the most active compounds contained a free hydroxyl group in their structures.

Three samples of ginger rhizomes obtained at different stages of vegetation were investigated in this study and found to exhibit similar activity in spite of one type being procured from ecological plantations (zo org), another from fresh and young (zo fr) plantations, and the last one from end of the vegetation period (zo tok) plantations. Although the differences were not found to be statistically significant, the volatile fraction from organic ginger was slightly more potent towards the inhibition of MAO-A enzyme, whereas older rhizomes were characterized by the lowest inhibitory activity. Also, Figure 4 presents the value of percentage inhibition of clorgyline—a known MAO-A inhibitor at a dose of 0.03 µM to compare the investigated samples to a known drug. The drug inhibitis the MAO-A enzyme in 96%.

In scientific databases, there is a multitude of literature on the MAO-A inhibitory activity of plant derived metabolites; however, only very few describe the application of the luminescence assay in the evaluation of their antidepressant properties via enzyme inhibition activity. Therefore, it is difficult to find suitable comparisons of our results with the previously published studies.

Zarmouh and co-workers [35] in their recent work described the inhibitory properties of an isoflavone—genistein in a bioluminescence assay. In their studies this compound at a concentration of 9.7 µM achieved 50% inhibition.

Peng and co-workers [29] reported that none of the isolated ginger phenolics possessed significant potential towards the inhibition of MAO-A compared to the references compound (curcumin and selegilline), although the total extract is widely known to have strong anti-depressant effect. In their study the maximal inhibitory potential did not exceed 30% for any of tested compounds. Thus they concluded that inhibition of MAO-A may represent only one potential mechanism of neuroprotective action of *Zingiber offcinale* rhizomas. The results obtained during the present study suggest that also lipophilic fraction of ginger have moderate activity towards the inhibition of monoamine oxidase, which may complement the findings of other studies. Moreover, these may also partially explain why the total extract is significantly more potent compared to isolated compounds and that the inhibitory activity is rather a resultant of a matrix effect.

According to the authors’ knowledge this is the first time the evaluation of the antidepressant potential of terpenes from ginger extracts is described. The results presented herein confirm the moderate potential of this group of secondary metabolites to act as MAO-A inhibitors. Moreover, the proposed protocol toward evaluating MAO-A inhibitory potential based on luminescence values can be a quick and simple tool for initial screening of natural products and guided isolation of active fractions and purified compounds as described in the present study.

## 3. Materials and Methods

### 3.1. Plant Material

2.5 kg of fresh ginger rhizomes (*Zingiber officinale* Roscoe) of Japanese origin (sample name: zo fr) were purchased in a local shop in Tokushima (Shikoku, Japan). According to the information included, they were cultivated on local plantations in Kochi and Tokushima prefectures in the summer 2013. First, the rhizomes were washed, finely cut and than used for the preparation of extract. A voucher specimen is stored in a freezer in the Chair and Department of Pharmacognosy with Medicinal Plants Unit at Medical University of Lublin, Poland. Also, 100 g of two different samples of ginger, each were purchased in a local supermarket in Tokushima for reference and they were given the following sample codes: zo org (from Japanese ecological plantations, Kochi, Japan) and zo tok (older rhizomes from local plantations) (see Figure 4).

### 3.2. Reagents

Reagents used for extraction, separation and chromatographic studies were of analytical grade and were purchased from Nacalai Tesque (Kyoto, Japan). These were as follows: *n*-hexane, diethyl ether, methanol, acetone, ethyl acetate. For the determination of MAO A inhibitory properties of both—the total extract, fractions from column 1 and the isolated terpenes a MAO-Glo^TM^ assay (Promega, Madison, WI, USA) was used. High purity deionized water (resistivity 18.2 MΩ cm^−1^) obtained using a Milli-Q water purification system (Millipore, Bedford, MA, USA) was used throughout this study.

### 3.3. Extraction Protocol

To obtain the extract for the separation and bioactivity studies, 2.0 kg of finely cut fresh ginger rhizomes were placed in two large stainless steel containers and mixed with 2 L of *n*-hexane, each. The containers were shaken for 24 h and later the solvent was filtered out and evaporated to dryness on a rotary evaporator at 35 °C under reduced pressure. A fresh portion of *n*-hexane (1 L) recycled from the previous step was poured on to the rhizomes and shaken for the next 24 h. The procedure was repeated three times. The filtrates were combined and evaporated to dryness. Finally 300 g of resinous extract was obtained. The ready extract and the fractions obtained in the following steps were kept in the fridge in tightly closed containers at 4 °C in between the measurements or separations, whereas the unprocessed rhizomes were stored in a freezer for eventual repetitions and other determinations.

### 3.4. Fractionation of the Extract

The obtained *n*-hexane extract from ginger rhizomes was carefully fractionated by column chromatography using normal phase silica gel. Similar fractions were joined and tested for their MAO-A inhibitory activity using a MAO-Glo^TM^ assay. Three most promising collective fractions were further purified by semi-preparative HPLC chromatography or using gravity columns to deliver single terpenes for further evaluation in the same assay (see Figure 3).

First 5 g of *n*-hexane extract mixed and dried with 5.2 g of silica depot was introduced on the top of a glass column (internal diameter of 4 cm) packed with 250 g of silica gel washed with *n*-hexane (column 1). 169 fractions were obtained from a gradient of diethyl ether (EtOEt) in *n*-hexane. 100% of *n*-hexane (100 mL) applied at the very beginning provided fraction 0, 1% EtOEt (100 mL)—resulted in fraction 1, 2% EtOEt (200 mL)—in fractions 2–8, 3% EtOEt (200 mL)—in fractions 2–8, 4% EtOEt (200 mL)—fractions 19–27, 6% EtOEt (100 mL)—fractions 28–32, 7% EtOEt (200 mL)—fractions 33–41, 10% EtOEt (300 mL)—fractions 42–52, 12% EtOEt (400 mL)—fractions 53–72, 14% EtOEt (200 mL) —fractions 73–81, 15% EtOEt (300 mL)—fractions 82–94, 17% EtOEt (300 mL)—fractions 95–108, 18% EtOEt (200 mL)—fractions 109–118, 20% EtOEt (400 mL)—fractions 119–136, 25% EtOEt (400 mL)—fractions 137–143, 30% EtOEt (500 mL)—fractions 151–173, 35% EtOEt (600 mL)—fractions 174–207, 45% EtOEt (200 mL)—fractions 208–216, 95% EtOEt (200 mL)—fractions 217–231. At the end the column was washed with 100 mL of acetone.

The obtained fractions were air dried under the fume hood and their composition was assessed by TLC chromatography. Similar fractions were joined. The visualization of TLC plates was performed using methanolic solution of anisaldehyde prepared according to the European Pharmacopeia. Additionally, the composition of all collective fractions was controlled by GC-MS spectrometer in a further described method.

#### 3.4.1. Semi-Prepartive HPLC Purification of Fraction 32–38

A semipreparative normal phase column Cosmosil 5SL-II 250 mm × 10 mm, d = 4.6 µm (Nakalai Tesque Inc., Kyoto, Japan) was used for the purification of three identified major terpenes from fraction 32–38 of column 1. Prior to the analysis 82 mg of fraction 32–38 was dissolved in 1 mL of *n*-hexane and 20 µL of the solution was injected on a chromatography column each time. The purification was performed on a Jasco chromatograph composed of a UV detector (JASCO UV-970, Easton, MD, USA) and a binary pump (JASCO PU-980, Easton, MD, USA) using the following gradient of diethyl ether (solvent A) in *n*-hexane (solvent B): 0 min—20% of A in B, 20 min—80% of A in B, 35 min—80% of A in B. The flow rate was 3 mL/min, the analysis time—60 min, the detection wavelength: 235 nm, and the temperature of separation: 25 °C. 5 mL fractions were collected throughout the analysis. All of them were subjected to TLC analysis of composition, similar fractions were joined and analyzed by GC-MS for the purity control.

#### 3.4.2. The Application of Silver Nitrate Impregnated Silica Gel in the Purification of Terpenes from Fractions 1–20 and 52–58

As it was difficult to obtain significant quantities of terpenes in HPLC separations, a gravity column filled with silica gel impregnated with silver nitrate was used, according to the previously published methodology [34]. 50 g of silica gel was transferred to a round-bottomed flask and poured with 30 mL of 25% solution of silver nitrate. The mixture was evaporated until dryness under reduced pressure in a rotary evaporator at 40 °C. Later the adsorbent was dried in an electrical oven in the temperature of 110 °C to remove water completely. The so prepared stationary phase was kept in the brown glass bottle (protection form the UV) and then transferred to a thin glass column and flushed with a 2% solution of EtOEt in *n*-hexane (column 2). Then 211 mg of the fraction 1–20 from column 1 was mixed with 2 g of silica depot and introduced on the top of the column 2. 2% solution of EtOEt in *n*-hexane was used to develop the column 2 under pressure (800 mL). 

A similar column (column 3) was prepared for the purification of metabolites from fraction 52–58. 428 mg of the sample was mixed with 3 g of silica depot and developed on the column by 2% solution of EtOEt in *n*-hexane (1000 mL). At the end the column was flushed with 80% EtOEt in *n*-hexane (200 mL).

TLC chromatograms (NP Silica Gel TLC plates, Merck, Darmstadt, Germany) were prepared to determine the composition of fractions and isolated compounds. The TLC plates were developed in different ratio of diethyl ether in *n*-hexane, depending on the polarity of metabolites—from 15% of diethyl ether in *n*-hexane (fractions 1–54), through 25% for fractions 62–133 until 29% of diethyl ether in *n*-hexane (fractions 136–220).

### 3.5. GC-MS Analysis of the Obtained Extract and Fractions

The obtained fractions were left to dry and than they were resuspended in 0.5 mL of *n*-hexane, filtered through a nylon membrane filter (pore diameter of 0.45 µm) and subjected to GC-MS analysis. 10 µL of each sample was directly injected to a gas chromatograph (GC) (Agilent Technologies 4890N, Santa Clara, CA, USA) equipped in a capillary column HP-5MS (30 m, 0.25 mm × 0.26) and in an MS detector (Agilent Technologies 5973, Santa Clara, LA, USA) using a Hamilton syringe. The analysis was performed in a temperature-dependant method according to the previously published methodology [32]. Briefly, the inlet temperature was set at 250 °C, the initial temperature at 50 °C, the final temperature at 250 °C, and the heating ramp was set as 5 °C/min. The initial and final temeratures were held for 3 and 15 min, respectively. The helium flow was set at 1 mL/ min. The identification of isolated terpenes was performed based on the comparison with scientific literature, GC-MS libraries, co-injected standards of hydrocarbons’ solution, and in relation to the obtained retention indices. First, the total extract was injected on the GC-MS spectrometer and each major metabolite was identified in relation to the previously injected solution of hydrocarbons, which helped to determine the quantity of carbon atoms in each of the analyzed terpenes. The structure suggestions from the GC-MS libraries were sorted according to their similarity to the experimental spectra. Also, the retention indices of the tentative structures were carefully analyzed to provide a continuous increase in their values throughout the chromatograms. All identified metabolites were consulted with previously published literature results, also of the authors [32].

### 3.6. Monoamine Oxidase A (MAO-A) Assay

The inhibitory potential of samples against MAO-A enzyme (EC 1.4.3.4) was assessed using a MAO-Glo^TM^ test produced by Promega (Madison, WI, USA) (Valley et al., 2006) according to a protocol described elsewhere, with slight modifications [34,36]. Briefly, MAO substrate (a derivative of beetle luciferin) was dissolved in 105 µL of DMSO to obtain a 4 mm stock solution. Next 13.13 µL of the stock solution was diluted with MAO reaction buffer to a final volume of 328.25 µL (providing a 25-fold dilution of MAO substrate). Then, a homogenous solution of Reconstituted Luciferin Detection Reagent was prepared by mixing the Reconstitution Buffer with lyophilized Luciferin Detection Reagent. A series of MAO-A enzyme dilutions in MAO reaction buffer were prepared (2-, 10-, 100- and 1000-fold) to evaluate the highest intensity of luminescence. The 100-fold dilution was chosen as the best among all the others and this concentration of MAO-A enzyme was used in all further determinations. To measure the inhibitory properties of the samples, MAO substrate (12.5 µL) was mixed with 12.5 µL of the test compound or extract at the concentration of 125 µg/mL. This particular concentration was selected in the initial tests performed on the total extract among 25, 50, 100 and 125 µg/mL as the most promissing one. To initiate the reaction, 25 µL of MAO-A enzyme (100-fold dilution) was added and mixed briefly. For negative control reactions 25 µL of MAO reaction buffer was added instead of an enzyme. The tubes were incubated in a room temperature for 1 h and subsequently 50 µL of Reconstituted Luciferin Detection Reagent was added and mixed immediately. The samples were incubated at room temperature for 20 min to generate and stabilize the luminescent signal. Blank samples were prepared by replacing the tested samples by the MAO reaction buffer. To avoid solvent’s influence on MAO enzyme required the corrections were made (the luminescence signal was measured according to the same protocol, where instead of the sample only specific volume of the solvent was used). Net MAO-dependent luminescence (net RLU) was calculated by subtracting the average luminescence of the negative control reactions (without MAO enzyme) from that of the MAO-containing tests. The net signal from blank reactions represented the total MAO-A enzyme activity and was treated as the control in the performed measurements (see Figure 4). This value was accepted as 100%, which was equal to net RLU of 18.5 × 106. Differences between the average net signal from total MAO activity and the net signals from reactions with test compounds or extract represents the inhibitory potential of the tested compounds toward MAO-A enzyme and these were expressed as percentage activity. All determinations (including negatives and blanks) were performed in triplicates using a GloMax 20/20n luminometer produced by Promega (Madison, WI).

## 4. Conclusions

The performed bioguided fractionation of *n*-hexane extract from ginger rhizomes confirmed the ability of terpenes present in the plant to inhibit the MAO-A enzyme activity. Among the most active components of the studied fractions geraniol, terpinen-4-ol and 1,8-cineole were confirmed with percentage inhibition of MAO-A equal to 44.1%, 42.5%, and 41.1%, respectively.

## Figures and Tables

**Table 1:** The GC-MS identification data on volatile components present in three collective fractions from column 1 and their content in three studied total extracts.

Compound	Rt (min)	Retention Index (RI)	No of Scan	Zo Tok (%)	Zo Fresh (%)	Zo Org (%)
ƴ-Terpinen	12.48	1133	352	2.57	3.81	4.71
1,8-Cineol	12.90	1141	368	0.26	0.82	1.18
α-Citronellal	16.55	1291	549	0.29	0.11	0.28
Borneol	16.94	1306	567	0.63	0.55	1.02
Terpinen-4-ol	17.31	1328	584	0.22	0.08	0.27
Geraniol	19.70	1420	697	3.46	5.35	0.41
Geranial	20.10	1438	717	12.41	5.66	7.99
Isobornyl acetate	20.49	1455	736	0.28	0.16	0.19
Geranyl acetate	23.13	1575	863	5.05	5.90	0.35
α-Zingiberene	26.10	1716	1002	22.22	30.53	39.53
(*E*,*E*)-α-Farnesene	26.34	1731	1016	12.49	16.28	9.27
β-Sesquiphellandrene	26.76	1750	1034	8.47	10.48	13.41

## Supplementary Materials

The following are available online. Table S1: The percentage composition of collective fractions and the purity of the finally obtained terpenes, Figure S1: The structures of major terpene constituents of *Zingiber officinale* oleoresin.
