# Supplementary material for: MAO-A Inhibitory Potential of Terpene Constituents from Ginger Rhizomes—A Bioactivity Guided Fractionation
Source: Molecules. 2018 May 29;23(6):1301. doi: 10.3390/molecules23061301 (PMC6099963; doi:10.3390/molecules23061301)
Supplement: Supplementary file 1 [file molecules-23-01301-s001.pdf]

SUPPLEMENTARY MATERIAL

MAO-A Inhibitory Potential of Terpene Constituents from Ginger Rhizomes—A Bioactivity Guided Fractionation

Wirginia Kukula-Koch [1,*], Wojciech Koch [2], Lidia Czernicka [2], Kazimierz Głowniak [1,3], Yoshinori Asakawa [4], Akemi Umeyama [5], Zbigniew Marzec [2] and Takashi Kuzuhara [6]

[1] Chair and Department of Pharmacognosy with Medical Plant Unit, Medical University of Lublin, 1 Chodźki Str., 20-093 Lublin, Poland; kglowniak@pharmacognosy.org
[2] Chair and Department of Food and Nutrition, Medical University of Lublin, 4a Chodźki Str., 20-093 Lublin, Poland; kochw@interia.pl (W.K.); lidia.czernicka@umlub.pl (L.C.); zbigniew.marzec@umlub.pl (Z.M.)
[3] University of Information Technology and Management in Rzeszów, Department of Cosmetology, Kielnarowa 386a, 36-020 Tyczyn, Poland
[4] Department of Pharmaceutical Chemistry, Faculty of Pharmaceutical Sciences, Tokushima Bunri University, Yamashiro-cho, Tokushima 770-8514, Japan; asakawa@ph.bunri-u.ac.jp
[5] Department of Pharmacognosy, Faculty of Pharmaceutical Sciences, Tokushima Bunri University, Yamashiro-cho, Tokushima 770-8514, Japan; umeyama@ph.bunri-u.ac.jp
[6] Laboratory of Biochemistry, Faculty of Pharmaceutical Sciences, Tokushima Bunri University, Yamashiro-cho, Tokushima 770-8514, Japan; kuzuhara@ph.bunri-u.ac.jp
* Correspondence: virginia.kukula@gmail.com; Tel.: +48-81-448-7087

	collective fractions 1-20	collective fractions 32-38	collective fractions 52-54	Purity [%]
mass	**211 mg**	**284 mg**	**500 mg**	
composition [%]				
γ-Terpinen			0.48%	92.90
1,8-Cineol		20.70%		95.40
α-Citronellal			1.38%	97.50
Borneol				96.10
Terpinen-4-ol			1.49%	95.90
Geraniol			6.74%	97.80
Geranial			11.10%	94.30
Isobornyl acetate		19.48%		98.30
Geranyl acetate		51.40%		95.90
α-Zingiberene	42.0%			97.10
(E.E)-α-Farnesene	20.3%			96.20
β-Sesquiphellandrene	9.11%			97.70

Table S1. The percentage composition of collective fractions and the purity of the finally obtained terpenes .

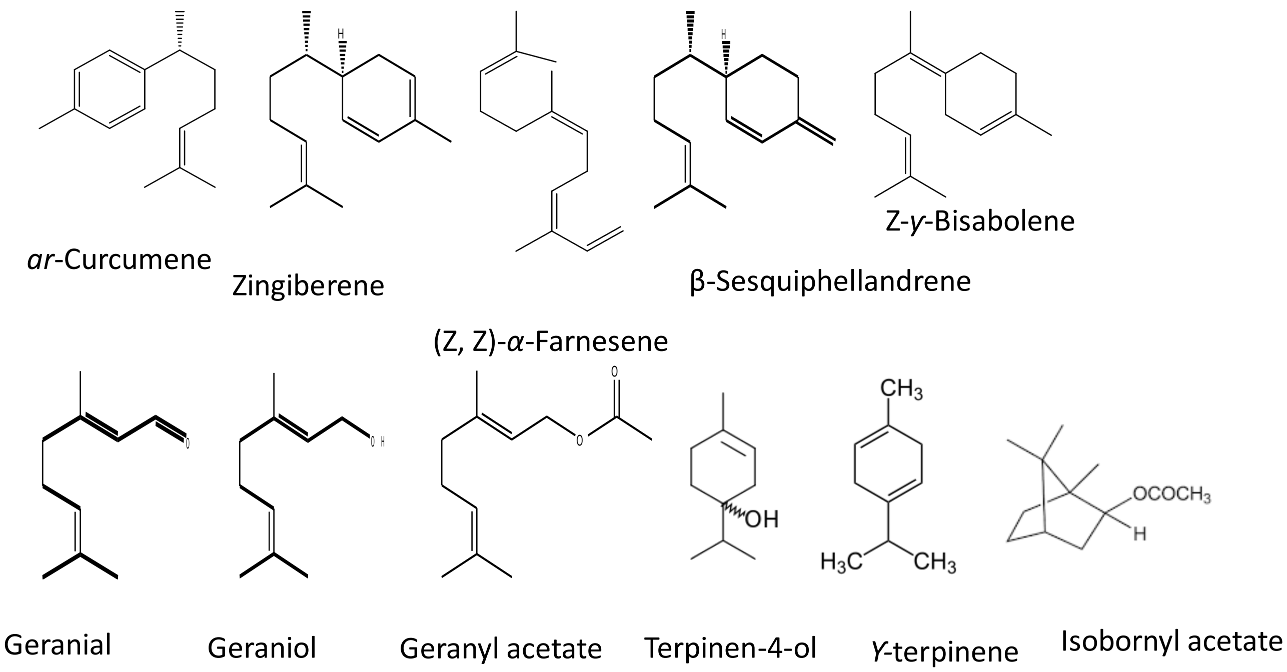

Figure S1. The structures of major terpene constituents of *Zingiber officinale* oleoresin.
